# Development of a compact superconducting rotating-gantry for heavy-ion therapy

**DOI:** 10.1093/jrr/rrt205

**Published:** 2014-03

**Authors:** Yoshiyuki Iwata, K. Noda, T. Murakami, T. Shirai, T. Furukawa, T. Fujita, S. Mori, S. Sato, K. Mizushima, K. Shouda, T. Fujimoto, H. Arai, T. Ogitsu, T. Obana, N. Amemiya, T. Orikasa, S. Takami, S. Takayama

**Affiliations:** 1 National Institute of Radiological Sciences, Chiba, Japan; 2 AEC, Chiba, Japan; 3 KEK, Ibaraki, Japan; 4 NIFS, Gifu, Japan; 5 Kyoto University, Kyoto, Japan; 6 Toshiba Corp., Tokyo, Japan

**Keywords:** heavy-ion therapy, rotating gantry, superconducting magnet, scanning irradiation

## Abstract

An isocentric superconducting rotating-gantry for heavy-ion therapy is being developed [ 1]. This rotating gantry can transport heavy ions having 430 MeV/u to an isocenter with irradiation angles of over ±180°, and is further capable of performing fast raster-scanning irradiation [ 2]. A layout of the beam-transport line for the compact rotating-gantry is presented in Fig. 1. The rotating gantry has 10 superconducting magnets (BM01-10), a pair of the scanning magnets (SCM-X and SCM-Y) and two pairs of beam profile- monitor and steering magnets (ST01-02 and PRN01-02). For BM01-BM06 and BM09-BM10, the combined-function superconducting magnets are employed. Further, these superconducting magnets are designed for fast slewing of the magnetic field to follow the multiple flattop operation of the synchrotron [ 3]. The use of the combined-function superconducting magnets with optimized beam optics allows a compact gantry design with a large scan size at the isocenter; the length and the radius of the gantry will be to be ∼13 and 5.5 m, respectively, which are comparable to those for the existing proton gantries. Furthermore, the maximum scan size at the isocenter is calculated to be as large as ∼200 mm square for heavy-ion beams at the maximum energy of 430 MeV/u.

All of the superconducting magnets were designed, and their magnetic fields were calculated using the Opera-3d code [ 4]. With the calculated magnetic fields, beam-tracking simulations were made. The simulation results agreed well with those of the linear beam-optics calculation, proving validity of the final design for the superconducting magnets. The five out of 10 superconducting magnets, as well as the model magnet were currently manufactured. With these magnets, rotation tests, magnetic field measurements and fast slewing tests were conducted. However, we did not observe any significant temperature increase, which may cause a quench problem. Further, results of the magnetic field measurements roughly agreed with those calculated by the Opera-3d code.

The design study as well as major tests of the superconducting magnets was completed, and the construction of the superconducting rotating-gantry is in progress. The construction of the superconducting rotating-gantry will be completed at the end of FY2014, and be commissioned within FY2015.

**Fig. 1.**
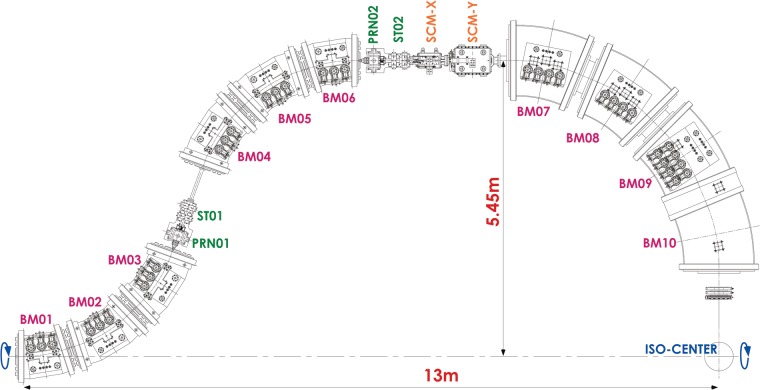

Layout of the superconducting rotating-gantry. The gantry consists of 10 superconducting magnets (BM01–BM10), a pair of the scanning magnets (SCM-X and SCMY), and two pairs of beam profile-monitor and steering magnets (STR01–STR02 and PRN01–PRN02).
